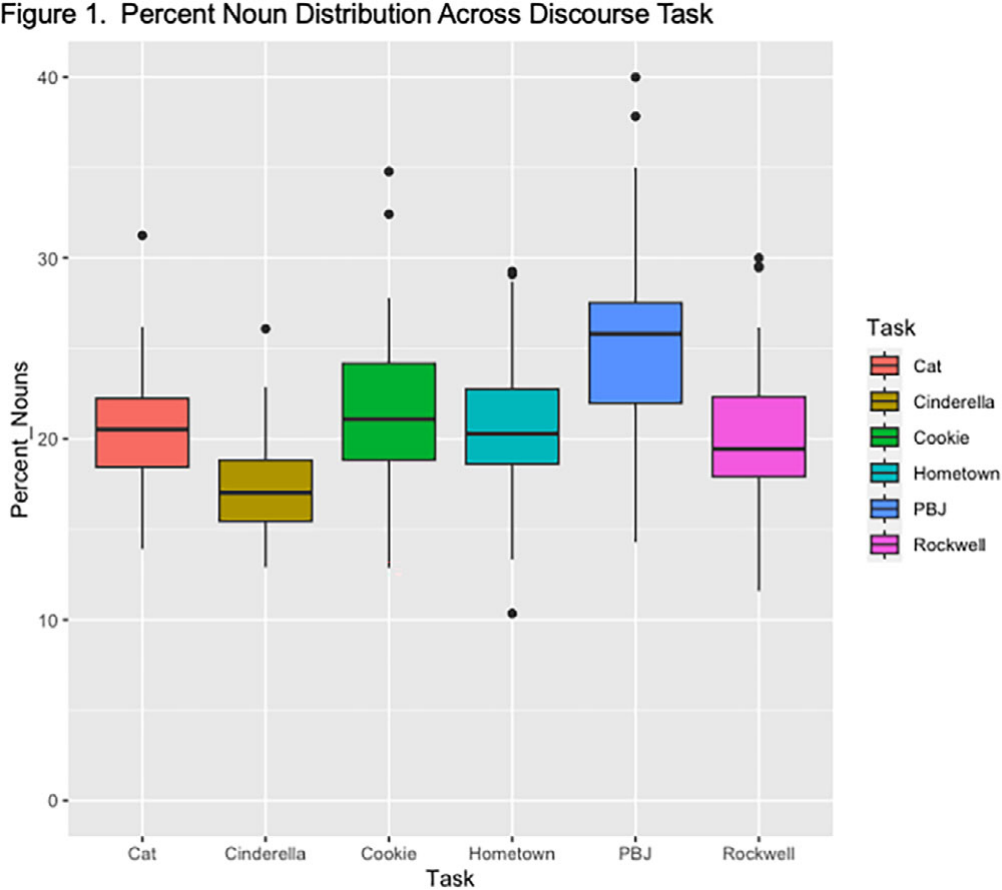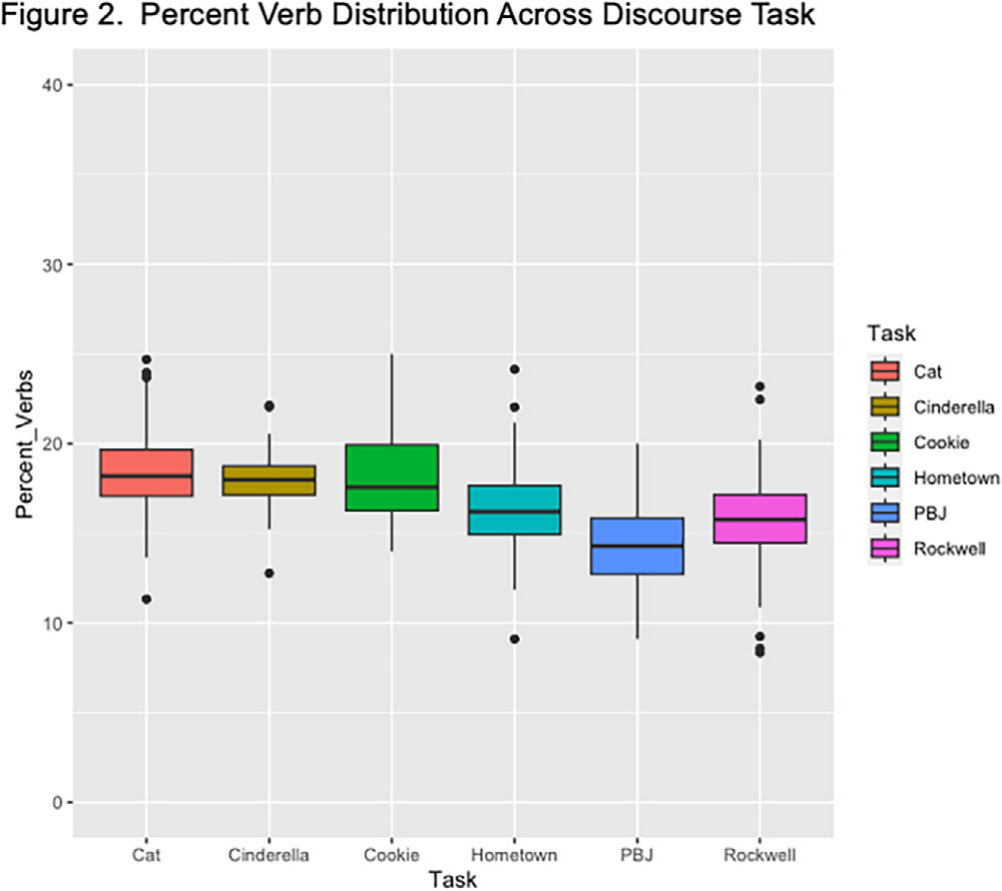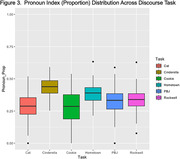# Use of Technology to Remotely Assess Language as a Non‐Invasive Biomarker: The Importance of Language Task

**DOI:** 10.1002/alz.084018

**Published:** 2025-01-03

**Authors:** Anna K Saylor, Matthew L. Cohen, Brian MacWhinney, Davida Fromm, Faith Stagge, Alyssa M Lanzi

**Affiliations:** ^1^ University of Delaware, Newark, DE USA; ^2^ Carnegie Mellon University, Pittsburgh, PA USA

## Abstract

**Background:**

There is growing evidence that discourse (i.e., connected speech) could serve as a cost‐effective and ecologically valid means of identifying individuals with prodromal Alzheimer’s disease. Specifically, metrics of semantic content appear to be sensitive to early mild cognitive impairment (MCI; Mueller, Koscik, Clark et al., 2018). These results were from spoken discourse samples from a brief, decontextualized picture description task (i.e., “Cookie Theft”) that may have task‐specific language demands. This study aims to determine if discourse produced from other task types elicits different levels of semantic content.

**Method:**

Discourse data were taken from the Delaware Corpus in DementiaBank, an open‐access online database of multimedia interactions (Lanzi et al., 2023). Participants’ cognitive status was documented in the database: cognitively healthy (n = 24) or MCI (n = 43) based on NIA‐AA and Petersen’s (2011) criteria. Using Zoom, participants completed six spoken discourse tasks including picture description (“Cookie Theft”), story narrative (“Cat Rescue”, “Rockwell”, “Cinderella”), procedural discourse (“PBJ”), and personal narrative (“Hometown”). Discourse samples were transcribed using an automatic speech recognition (ASR) pipeline and analyzed using CLAN software (Liu et al., 2023). Semantic content was measured by three variables: percent nouns, percent verbs, and pronoun index (Mueller, Koscik, Hermann et al., 2018).

**Result:**

Interaction effects of cognitive status by task were evaluated for each semantic variable. Two‐way ANOVAs yielded non‐significant interaction effects (p>0.05). Therefore, we aggregated our task data across cognitive status to conduct one‐way ANOVAs. Each semantic variable ANOVA produced a main effect of task: F_%noun_(5, 394) = 30.63; F_%verb_(5, 391) = 31.64; F_pronoun_(5, 391) = 23.68, all *p value*s<.001 (see figures). Post‐hoc comparisons revealed that the highest level of each semantic variable was produced by a different discourse task (e.g., PBJ = highest percent nouns).

**Conclusion:**

This discourse study was efficiently executed with the use of remote data collection, an ASR transcription pipeline, and CLAN for analysis. Each discourse task elicited differing levels of semantic content. As efforts continue to study language as a non‐invasive biomarker for cognitive decline, researchers should consider which discourse task(s) would best support their aims.